# Implementation of the physical function ICU test tool in a resource constrained intensive care unit to promote early mobilisation of critically ill patients- a feasibility study

**DOI:** 10.1186/s40945-016-0026-0

**Published:** 2016-10-19

**Authors:** Cathrine Tadyanemhandu, Shamila Manie

**Affiliations:** 1 Department of Rehabilitation, College of Health Sciences, University of Zimbabwe, PO Box AV 178. Avondale, Harare, Zimbabwe; 2 Department of Health and Rehabilitation Sciences, Faculty of Health Sciences, Division of Physiotherapy, University of Cape Town, Anzio Road, Observatory, Cape Town, South Africa

**Keywords:** Early mobilisation, Feasibility, Functional status, Outcome measures, Evidence-based practice

## Abstract

**Background:**

The shift of focus in outcome measures from mortality to assessment of functional status in intensive care unit (ICU) patients has resulted in the emergence of mobilisation of critically ill patients as a standard physiotherapy practice in most medium and high income countries. The aim of this study was to determine the feasibility of an early mobilisation program and to report on the changes in patient clinical outcomes following the intervention in a low income country.

**Methods:**

A prospective cohort study was carried out at one public hospital. An adult cohort of 35 patients was recruited within 24 h of being admitted into the unit, irrespective of ventilation method over a period of three months. An early mobilisation programme was implemented and prescribed using the Physical Function ICU Test (PFIT-s) which commenced in either the ICU or high dependent unit.

**Results:**

The median age of the 35 patients was 29 years (IQR = 24–45 years). More than half of the patients had undergone surgery due to either gastrointestinal problems or obstetrical complications. A total of 94 out of a possible of 219 exercise sessions were delivered to the patients (43.0 %). The tool was implemented in 32 (91.4 %) patients on the initial PFIT-s measurement and 16 (45.7 %) of the patients required the assistance of two people to stand. The Initial PFIT-s mean score was 5.3 ± 1.8. On final PFIT-s measurement, out of the 30 (85.7 %) patients seen, 15 (42.9 %) of the patients did not require any assistance to stand and the final PFIT-s mean score was 7.0 ± 1.9. There was a significant difference in both the initial PFIT-s total score (*t-value* = 2.34, *df* = 30, *p* = .03) and the final PFIT-s score (*t-value* = 3.66, *df* = 28, *p* = .001) between males and females. During the treatment, no adverse event occurred in any of the patients.

**Conclusion:**

An early mobilisation program using PFIT-s was feasible and safe. There was a difference in functional capability based on gender, with males being more functionally active. Specific inclusion and exclusion criteria can lead to a delayed early mobilisation activities in ICU patients.

**Trial registration:**

Pan African Clinical Trials Registry PACTR201408000829202. Registered 15 August 2014.

## Background

Management of critically ill patients has traditionally involved periods of immobility, bed rest, use of analgesics and sedative medication [1]. This traditional model of practice has been geared to promote patient comfort, safety, respiratory synchrony and mechanical ventilatory support for severe respiratory failure [2]. Physiotherapy plays a pivotal role in the rehabilitation of intensive care unit (ICU) patients [3]. The international traditional physical therapy recommendations according to the European practice in ICU include suctioning, percussions, vibration, the application of passive range of movements and encouragement of an active range of movement early in the ICU stay [4]. The major focus of physiotherapy treatment in ICU has been management of respiratory complications such as retained pulmonary secretions, atelectasis and the avoidance of re-intubation [5, 6]. Common problems which are increasingly being recognised in patients because of this mode of intervention include post intensive care syndrome which describes new or worsening problems in physical, cognitive or mental health status arising after a critical illness and persisting beyond acute care rehabilitation [7–10]. The syndrome referred to as intensive care unit acquired weakness (ICU-AW) has been found to develop in ICU patients [11]. The actual immobility which is experienced by patients in ICU has been found to play a major role in the development of the ICU-AW through the decreased muscle protein synthesis and proteolysis [12]. The cause and frequency of this weakness during or immediately after ICU admission has been attributed to a period of enforced bed rest. Prolonged immobility is harmful with rapid reductions in muscle mass, bone mineral density and impairment in other body systems evident within the first week of bed rest, which is further exacerbated in individuals with critical illness [13]. Both CIP and CIM have been reported to delay weaning, compromising rehabilitation, leading to increased hospital and ICU morbidity and mortality [2, 7, 14]. The weakness has also been found to be associated with adverse long-term outcomes like functional status and quality of life of these patients [2, 7, 14]. Knowledge of the effects of prolonged bed rest on multiple body systems and its association with long-term outcomes of the patients has resulted in changes in standard clinical practice [13, 15].

Early mobilisation of critically ill patients, although not new, is an ICU intervention that is beginning to receive significant attention by ICU multidisciplinary team and positive results on patients’ outcomes after discharge is being reported from reviews done [16–18]. Previous studies in the subject of early mobility had reported potential role of rehabilitation to improve patient outcomes through decreasing duration period on mechanical ventilator, length of stay in the ICU and improved functional status at discharge but the studies have been done in either high-income or medium-income countries [15, 19–22]. The greatest burden in providing critical care services in a low income country like Zimbabwe is mainly due to the high shortage of equipment and the high demand for ICU beds [23, 24]. Infection, trauma, post-operative treatment and perinatal complications are much more common causes of admission to an ICU in low income countries than complications of chronic cardiac, vascular and pulmonary diseases which are prevalent in high to medium income countries [24–27]. Hence, because of the stated causes of ICU admission, a much younger population, which will be the most active group is mostly admitted in a low income country ICU. Therefore, there is a great need to investigate the practicality of early mobilisation intervention in populations with different settings to determine its potential to meet the intervention’s goals [28].

The economy of Zimbabwe declined rapidly from the late 1990s and this directly contributed to a deterioration of health infrastructure, loss of health professionals, drug shortages and a decline in the quality of public health services [29]. The presence of endemic diseases such as HIV and trauma in the low-income countries present a substantial burden to health care systems [24, 27]. Although the incidence rate of HIV infection among adults has been reported to be declining in Zimbabwe, the epidemic remains significantly high; representing the third largest burden in Southern Africa [30]. Additionally, Hanekom, Faure & Coetzee [31] argue that there is need to evaluate the effects of any physiotherapy treatment in a unique environment since it is not clear whether the specific health characteristics of the population will affect patient’s outcome from ICU. According to a study done, it showed that there was no form of mobilisation of patients out of bed before ICU discharge and physiotherapists in Zimbabwe are still relying on chest physiotherapy as a treatment option in ICU and not considering functional activities as early as possible to improve patient outcomes [32].

In light with this, it was found necessary to implement an early mobilisation intervention which was found to be the current best practice in ICU from literature. The study was done to determine the feasibility of an early mobilisation program using the interval-scored Physical Function ICU test (PFIT-s) [33] tool with regard to

- Number of eligible patients
- Assessing the potential for successful implementation
- Practicality of delivering the treatment in the setting in terms of resources needed
- Change in outcome measures relating to physical functioning as measured by PFIT-s

## Methods

### Study design and study procedures

A prospective cohort study was carried out at a teaching, public hospital in Harare. The hospital is the largest and most sophisticated public hospital complex in the country and has a capacity of one thousand eight hundred beds with the largest intensive care unit which is a six-bed unit and a five-bed high dependency unit (HDU). The study was approved by the University of Cape Town, Human Research Ethics Committee (REF; 190/2012), Medical Research Council of Zimbabwe (MRCZ/B/342), Joint Research Ethics Committee (JREC/179/12) and the Institutional Review Board of the hospital. Patients were recruited from the mixed adult medical-surgical ICU and HDU from 20 November 2012 to 19 February 2013. All patients who were recruited following the inclusion criteria provided written informed consent or had their next of kin consent on their behalf to participate in the study. The study was registered with the Pan African Clinical Trials Registry (PACTR201408000829202) on the 15th of August 2014 (www.pactr.org).

Using the Statistica Version 13 sample size calculation, the sample size was calculated using the pre and post PFIT-s scores before and after weaning from a mechanical ventilator from the study done by Skinner et al. [34]. Using the marching on the spot component, the number of steps done, it was anticipated that a sample size of 37 patients would be required to detect a significance difference (*p* = .05) with a power of 80 % if the pre scores before weaning from mechanical ventilator was 38 steps and that after weaning from the mechanical ventilator was 124 steps using a standard deviation of 129 steps.

Inclusion criteria included all patients who were admitted to ICU and High dependency unit (HDU) irrespective of ventilation method (non-invasive or invasive), aged 18 years old or older, medically stable. Mobilisation of patients was initiated based on a priori selected [19] that included:

- ➢ Neurological criterion- the patient had to be awake which was assessed by response to verbal stimulation using the De Jonghe 5-point command for alertness [11]. The sedation management of patients depended on the ventilator mode. The patients who were on pressure or volume limited modes of ventilation were on continuous bolus of sedation and interruption of sedation was only done for patients being weaned off the ventilator.
- ➢ Respiratory Criterion- a fraction of inspired oxygen (FiO_2_) of less than or equal to 0.6 and positive end-expiratory pressure (PEEP) of less than or equal to 8 cm of water.
- ➢ Circulatory criterion- the absence of orthostatic hypotension and catecholamine drips.

Exclusion criteria included all patients who had one of the following;

- ➢ Cognitive impairment defined as having trouble remembering, learning new things, concentrating or making decisions that affect everyday life, before admission into the critical care unit
- ➢ Inability to walk without assistance of a walking device for any distance before acute illness
- ➢ Neuromuscular disease that could impair weaning (Myasthenia gravis, Guillain-Barre), acute stroke, head injury
- ➢ Hip fracture, unstable cervical spine and pathologic fracture
- ➢ Any patient the treating intensive care unit team advised on avoiding mobilisation for any other reason not aforementioned.

### Data collection

The data which was recorded on the questionnaire included; demographics data which include age, gender and diagnosis; Clinical outcomes which include method of ventilation, ventilator settings, duration on mechanical ventilation and length of stay in the unit, the number of days physiotherapy treatment was received, PFIT-s components (assistance needed to come to standing, time to march on spot and the number of steps done, muscle strength using Oxford Scale) and vital signs measured pre and post treatment. The vital signs which were looked at included heart rate, blood pressure, mean arterial pressure, oxygen saturation and respiratory rate. Adverse events as a result of the mobilisation were also documented prospectively.

### Intervention

The patients were recruited to participate in the study within the first 24 h of admission into the ICU or HDU. The program was implemented and prescribed using a specific inclusion and exclusion criteria described above. The modified version of PFIT-s which has the following components; sit to stand level of assistance, marching on the spot cadence (Steps/min), muscle strength (knee extension and shoulder flexion) which provide a total score out of 10 [33, 35] was used to prescribe exercises as well as an outcome measure of functional capacity at discharge. An ordinal score is first obtained out of 12 (ie. adding scores out of 3 for the 4 domain items on the tool). The PFIT-s (interval score) is then obtained from the Rasch analysis algorithm table. The conversion to an interval scoring scale is required in order for parametric statistics to be used in data analyses [33].

The PFIT-s was used from the first day of inclusion to the study and the patient would start with the sitting component. Patients were sat over the edge of bed or in a chair, depending on the assessment of their level of functioning and also static balance in sitting and the level of assistance needed determined. The level of assistance needed was reassessed daily before marching on the spot. From sitting, the treatment would then progress to standing. Patients were then asked to practice sit to stand for five times either with or without assistance. Five times of sit to stand was done as a practice session for standing before patients could start marching on the spot. This was done in order for patients to get used to doing activities with the different attachments on them. After achieving this component, patients would then progress to marching on the spot and the number of steps and time taken to march were recorded for each day. The intensity of the exercises was prescribed based on the results from the initial day. Patients had to perform three repetitions of marching on spot for 70 % of their initial PFIT-s march on spot time based from the study by Skinner et al. [34]. Muscle strength testing for shoulder and knee were measured each day when the patient was back in a sitting position. PFIT-s was used each day up to discharge of the patient from ICU or HDU.

Respiratory and hemodynamic parameters were measured pre, during and post intervention in order to improve on quality. The parameters were also monitored during the intervention for any occurrence of adverse events since the patient was just connected to the monitor during the whole exercise time. The promote patient safety, the following were selected to be the adverse events [36] which required the exercise to be ceased and the attending registrar to be notified if any of those occured;

- Patient mean arterial blood pressure (MABP) less than 65 mmHg or greater than 120 mmHg and systolic pressure less than 90 mmHg and diastolic pressure less than 60 mmHg
- Patient heart rate of less than 50 or greater than 140 beats/min
- FiO_2_ greater than 0.6 and PEEP greater than 8 cm H_2_O
- Patient respiratory rate greater than 35 breaths/min
- A fall during transfer to the chair or walking
- Patient became pale and sweaty and/or the patient specifically requested to stop due to feeling acutely unwell

Physiotherapists in Zimbabwe work on a three month rotational basis covering a specific ward or area during the week [32]. As for the weekends, there is always a physiotherapist covering the critical care area and any other ward requiring the services. Hence this result in variability of the physiotherapist per each weekend as they also work on rotational basis. For the purpose of the study, the early mobilisation program was delivered by a research team of two physiotherapists (CT and TC) who are both qualified physiotherapists with more than five years working experience in the ICU and a highest level of qualification of Bachelors’ of Science Honours degree in Physiotherapy. The research team only worked in the ICU and HDU during the study period. The research team was first trained for fourteen days to be able to deliver the intervention by SM, who is a physiotherapy lecturer at University of Cape Town and she is well experienced in early mobilisation practice in ICU. A two day workshop on early mobilisation was done with a group of twenty physiotherapists from different hospitals in Zimbabwe. The first day focused on highlighting the benefits of early mobilisation by reviewing different studies done in other countries. The second day was a practical demonstration on how to mobilise ICU patients. SM was with the research team for the first 12 days of data collection, assisting with the exercise prescription. Chest physiotherapy was executed in all patients on mechanical ventilation before the mobility exercise by the research team. The chest physiotherapy comprised of percussions, vibrations and suctioning. The mobility intervention was administered to patients by the research team throughout their stay in the ICU and HDU, including weekends. The duration of each treatment session per patient varied daily based on the components the patients managed to complete on the day and also the number of steps done each day. The duration time ranged from 20 to 45 min.

### Statistical analysis

Patients’ data was analysed by intention to treat analysis and all patients recruited were included, regardless of whether they received the treatment up to discharge or they deceased before completing the final measurement. As the sample size was small, non-parametric statistics were used. Mann–Whitney U was used to compare the rank order in duration on mechanical ventilation, number of days physiotherapy treatment was received, the number of days before the mobilisation program started and length of stay in the unit between groups which were: males and females, discharged and deceased. The difference in means of cadence, initial and final PFIT-s score of males and females was tested using *t*-test. The Shapiro-Wilk test was used to assess for normal distribution. A *p* < 0.05 was accepted as statistically significant.

## Results

### Patients characteristics

A total of 52 patients were screened for eligibility over a period of three months, of which 35 met the inclusion criteria. The main reasons why the other 17 patients were excluded was because of unreduced fractures of the lower limbs and head injury patients with low Glasgow coma score. Of the 35 patients, 20 (57.1 %) were males. The median age of the patients was 29 with an interquartile range of 24–45 years, with most of the patients in the 20 to 30 age group. As shown in Table 1, of the patients who were admitted in the unit, the majority were under the department of general surgery and most of the patients were admitted from the operating theatre following an emergency surgery. The mortality rate was 14 % as five of the patients died during ICU stay.

**Table 1:** Characteristics of the patients (*n* = 35). All data are expressed as *n* (%) unless specified

	Frequency
Gender	
Males	20 (57.1)
Females	15 (42.9)
Age in years, median (IQR)	29 (24–45)
Location before admission	
Operating theatre	21 (60.0)
Transfer from another hospital	6 (17.1)
General ward	2 (5.7)
Casualty	2 (5.7)
Labour ward	4 (11.5)
Referring department	
Cardiothoracic	6 (17.1)
General surgery	16 (45.7)
Obstetrics and gynaecology	9 (25.7)
Medical	4 (11.5)
Diagnostic category of patient’s condition	
Non-surgical patients (Respiratory Disease)	2 (5.7)
Surgical patients	
Laparotomy for Gastrointestinal disorders	14 (40.0)
Thoracic and vascular	6 (17.1)
Thyroidectomy	1 (2.9)
Obstetrics and gynaecology	9 (25.7)
Polytrauma	3 (8.6)
Outcome	
Discharged	30 (85.7)
Deceased	5(14.3)

The majority of the patients had undergone laparotomy for gastrointestinal (GI) disorders and nine had surgery due to obstetrics and gynaecological problems. The most common cause of condition was infection followed by obstetrical complications and in few patients it was trauma and neoplasm.

### Clinical outcomes

The 35 patients spent a total number of 219 days in the ICU and HDU. The median length of stay of the discharged patients was four days (IQR = 2–7) and a range of 2–17 days, whilst for the deceased this period was nine days (IQR = 7–9) and a range of 1–15 days (Table 2). The median length of stay for females was five days (IQR = 2–9) whilst for males it was also five days (IQR = 2.5–8). There was no significant difference in length of stay for the discharged and deceased (*U* = 51.0, *Z adjusted* = −1.117, *p* = .26) and between males and females (*U = 147.5, Z adjusted = −0.067, p* = *.95)*.

**Table 2:** Clinical description of the patients’outcomes and reasons for delay in intervention

Description	Discharged	Deceased	Test
Duration on mechanical ventilation in days, median (IQR)	2 (1–5)	8 (3–9)	U = 39.0 *p* = 0.27
Length of stay in the unit in days, median (IQR)	4 (2–7)	9 (7–9)	U = 51.0 *p* = 0.26
Number of days prior to treatment, median (IQR)	1 (1–5)	8 (3–9)	U = 37.5 *p* = .08.
Reason for delay in intervention			
Sedated	13 (37.1)	1 (2.9)	
Cardiovascular instability	7 (20.0)	2 (5.7)	
Non delay in intervention	4 (11.4)		
Sedated and cardiovascular instability	4 (11.4)	2 (5.7)	
Respiratory distress	1 (2.9)		
Pain	1 (2.9)		
Number of days physiotherapy treatment was done, median (IQR)	3 (2–3)	0 (0–3)	U = 49.5, *p* = .22

On admission, 28 of the patients were put on mechanical ventilation while seven were on oxygen delivery through face mask. The median duration on invasive mechanical ventilation for 23 patients who were discharged was two days (IQR = 1–5, range = 1–13 days) whilst for deceased patients it was eight days (IQR = 3–9, range =1–9 days) (Table 2). There was no significant difference in the duration on mechanical ventilation for the discharged and deceased (*U* = 39.0, *Z adjusted* = −1.109, *p* = .27). Comparing duration period on mechanical ventilation and gender, females spent three days (IQR = 1–10) and males two days (IQR = 1–6) but there was no statistically significance difference (*U* = 88.5, *Z adjusted* = −0.333, *p* = .74) (Table 3).

**Table 3:** Comparison of the clinical outcomes and gender

Description	Males	Females	Test
Duration on mechanical ventilation in days, median (IQR)	2 (1–6)	3 (1–10)	U = 88.5 *p* = 0.75
Length of stay in the unit in days, median (IQR)	5 (2.5–8)	5 (2–9)	U = 147.5 *p* = 0.95
Number of days prior to treatment, median (IQR)	1 (1–5)	2 (1–6)	U = 131.5 *p* = .54
Number of days physiotherapy treatment was done, median (IQR)	3 (2–3)	2 (1–3)	U = 126.5 *p* = .43

A total of 94 out of a possible of 219 exercise sessions were delivered to the patients (43.0 %). The median number of days the early mobilisation programme was done for discharged patients was three (IQR = 2–3) days and a range of 1–9 days. In discharged patients, the median number of days for the delay in early mobilisation was one (IQR = 1–5) days and a range of 0–14 days (Table 2). The Mann–Whitney *U* test revealed that there was no significant difference in the rank order of the number of days before the mobilisation program started in discharged and deceased patients (*U* = 37.5, *Z adjusted* = −1.795, *p* = .08) and between males and females (*U = 131.5, Z adjusted = −0.6175, p = .54)* (Table 3).

Due to the stated inclusion and exclusion criteria, delayed activities were observed in the majority of the patients. Of the patients who were discharged, only four had early mobilisation physiotherapy from the first day they were admitted into the unit. Various reasons were cited why early mobilisation physiotherapy had to be delayed. Sedation was the reason why 13 (43.0 %) patients who were discharged had to delay participation in the early mobilisation programme, while 7 (23 %) patients delayed because they presented with cardiovascular instability (Table 2).

### Physical function ICU test

A total of 32 patients had the initial day of PFIT-s measurement whilst the other three patients did not get the initial measurement as they were sedated and did not manage to do any activity. On the initial day of PFIT-s measurement, 13 (37.1 %) of the patients managed to come up to standing and marched on the spot. Of these patients, seven were on face mask, three were on Pressure Support Mode and the last three were on Continuous Positive Airway Pressure mode. A total of five patients deceased, resulting in only 30 patients having the final PFIT-s measurement before discharge from the unit. The number of patients who marched on the spot improved to 24 (68.6 %) on the final PFIT-s measurement day. The assistance required to stand improved from two people at the initial PFIT-s measurement in 16 (45.7 %) patients to no assistance required on the final PFIT-s measurement in 15 (42.9 %) patients. The cadence also improved, comparing initial PFIT-s and the final PFIT-s results (Table 4). A significance difference was seen in cadence comparing the initial and final day of PFIT-s measurement (*p* = .001).

**Table 4:** The interval-scored Physical Function ICU Test measurements

Characteristic	Initial	Final	Statistics	*P* value
Assistance to stand (*n*;%)				
2 people	16 (45.7)	3 (8.6)		
1 person	11 (31.4)	12 (34.3)		
No assistance	5 (14.3)	15 (42.9)		
March on spot (mean;sd)				
Cadence	19.3 (24.5)	41.6 (22.7)	t-value = −5.17, df = 29	*p* = .001
Shoulder Flexion Grade 5, *n* (%)	6 (17.1)	16 (45.7)		
Knee extension Grade 5, *n* (%)	6 (17.1)	16 (45.7)		
Total PFIT-s Score	5.3 (1.8)	7.1 (1.8)	*t* = −3.55, *df* = 60,	*p* < .001

As the data was normally distributed, parametric tests were used to analyse the PFIT-s total scores (Shapiro-Wilk W = .95264, *p* = .171). There was a significant difference in the mean PFIT-s total scores of the patients on the two separate days of treatment (*t* = −3.55, *df* = 60, *p* < 0.001). The initial and final day the average PFIT-s total score was 5.3 (SD = 1.8) and 7.1 (SD = 1.8), respectively. There was a significant difference in the initial PFIT-s total score between males and females (*t-value* = 2.34, *df* = 30, *p* = .03) with males having a mean of 5.9 (SD = 1.6, range = 3.9–8.8) and females having a mean of 4.4 (SD = 1.9, range = 2.0–8.8). A significant difference in the final PFIT-s score between males and females was also observed (*t-value* = 3.66, *df* = 28, *p* = .001) with males having a mean score of 7.8 (SD = 1.4, range = 3.9–8.8) and females a mean score of 5.7 (SD = 1.8, range = 3.2–8.8).

The muscle strength of patients started on grade 2 of the Oxford Scale. On the final PFIT-s measurement, most patients had either a grade 4 or 5 in both their upper and lower limb. During the exercise treatment using PFIT-s, no adverse event occurred in any of the patients.

## Discussion

Our cohort had a median age of 29 years (IQR = 24–45), showing a trend of a young population being admitted into the ICUs in low to medium income countries [27, 31, 32, 37, 38]. The majority of patients were admitted into the ICU from the operating theatre after a laparotomy for gastrointestinal disorders and also obstetrical complications. This was also consistent with the stated reasons for ICU admission in low income countries which include mainly for postoperative management [24, 25]. This shows the study was conducted in a different cohort compared to the previous work involving PFIT-s because of the younger age of the population and the difference in diagnosis of the patients, with majority presenting with abdominal problems and obstetrical complications.

The PFIT-s as an outcome tool is a safe and inexpensive test of physical function with high clinical utility which has been found to be valid, responsive to change and predictive of key outcomes [33, 34]. The PFIT-s was chosen for our study because it is scored and consists of most of the components which have been reported in the literature to constitute early mobility programs which include: sitting over the edge of bed, marching on the spot, transfer to a chair and muscle testing [19, 39–42]. To our knowledge, the tool was developed in Australia and published studies on the tool are only from Australia and United States [33, 34, 43]. The tool was developed for the purpose of prescribing and evaluating rehabilitation in the critically ill and it has been found to be reliable and sensitive to detect change in the ICU population [33, 34].

The results of our study showed that early mobilisation of patients was feasible and safe in a Zimbabwean ICU setting. This was evident since 30 of the patients managed to perform the PFIT-s from admission until discharge. There was no occurrence of any adverse event during the mobilisation of the patients in our study and this was similar to results reported in other studies which were infrequent or no adverse events at all during early mobilisation [19–21, 34, 40].

For our study, the timing of mobilisation based on a priori criteria may appear conservative. The presence of a strict inclusion and exclusion criteria will result in limiting early mobilisation activities in some patients rather than implementing a detailed monitoring protocol of patient parameters during mobilisation [44]. Also in Brazil, a medium income country, mobilisation therapy in critically ill patients was safe and feasible however, in-bed exercises were the most prevalent activity and during mechanical ventilation, only a small percentage of activities involved standing or mobilizing away from the bed [45]. The number of the delivered exercise sessions in our study was lower than that reported by Skinner et al. [34]. The reasons might be due to the difference in the inclusion criteria, with the Australian cohort comprised of patients who had been admitted in the ICU for more than five days and where on tracheostomy as a weaning off measure. In our study, patients were enrolled within 24 h of admission into the unit and they were assessed to determine if PFIT-s could be initiated as soon as possible. The median days of delay of early mobilisation was one day with an interquartile range of 1–5 days. In our study, the definition of early used was the one given by Bailey et al. [19] which is, “*The interval starting with initial physiologic stabilisation and continuing through the ICU stay”*. In the study by Bailey et al., [19], they reported that early mobilisation started with a median of one day after discontinuation of catecholamine and a mean of 1.1 (SD = 2.1) days after discontinuation of sedatives. In a study done in Australia and New Zealand ICUs, of the 70 patients (36.5 %) who received early mobilisation during mechanical ventilation, the median (IQR) time from ICU admission to early mobilisation was five (3 to 8) days and the median (IQR) number of active mobilisation episodes per patient was 2 (1 to 4) [46]. However, in some studies early mobilisation started after a number of days due to the inclusion criteria. In a study by Nordon-Craft et al. [47], patients were recruited after a period of mechanical ventilation of four days or longer and baseline testing occurred at mean days of 15 (SD = 11).

Nonetheless, although early mobilisation was feasible, there were factors which were established which made the intervention difficult to execute early in this setting in some patients. The major barrier to early mobilisation in the cohort was sedation. The majority of the patients were sedated and this made the intervention difficult to execute. It was found that early mobilisation was difficult to initiate when patients were on pressure or volume limited modes of ventilation as they were on continuous bolus of sedation. Early mobilisation could only be initiated in patients who were being weaned off the ventilator or on Continuous Positive Airway Pressure (CPAP) and Pressure Support (PS) modes. The barrier of ongoing intravenous sedation was also highlighted by Bourdin et al. [47] as the most common contraindication to starting early rehabilitation in patients. Morris [48] argues that many hospitals have implemented general sedation protocol that includes some form of a daily awakening technique in order to promote early mobilisation. A strategy consisting of interruption of sedation and physical and occupational therapy in the earliest days of critical illness has been reported to result in better functional outcomes at hospital discharge [21]. However, interruption of sedation is not being practiced in Zimbabwe as yet and this imposed a major barrier in the implementation of the program in our study.

Several authors have reported that cardiovascular instability was a leading cause why early mobilisation could not be started in some patients [21, 40, 41]. Cardiovascular instability was the reason why some of the patients had to be treated in the afternoon after they had stabilised or the research team had to delay the initiation of the intervention until patient was stable. Other barriers to early mobilisation which have been highlighted in the literature but were not all common in our study included renal replacement therapy, out of ICU for a procedure, fatigue, patient refusal, lack of adequate equipment, morbid obesity, resistance to change, time constraints and costs [19, 42, 47, 48]. Of these aforementioned barriers, lack of adequate equipment was also applicable to our study as there were no mobile ventilators and a pulse oximeter to mobilise ventilated patients away from the bedside. The patients who were on mechanical ventilation only managed to march on the spot by the bed side.

The results of our study showed that there was an improvement in functional ability of the patients who managed to complete all the components of the PFIT-s on discharge. A reduction in the number of people required to assist patients to come up to standing by the final day of treatment was noted using the PFIT-s. This implied that majority of the patients at first were fully dependent on other people for them to be functionally active. Improvement was noted on the final PFIT-s measurement because more patients could come up to standing independently. Since this measurement was done before the patient was discharged from the unit, it showed the level of dependency of patients when finally discharged to the ward. It showed that the intervention was associated with promoting independence in patients before discharge from the unit. In the study by Skinner et al. [34], they reported that there was an improvement in assistance required before and after being weaned off the ventilator from two people to no assistance.

One advantage of the PFIT-s is that even though the patient might be unable to complete all the components, the score which is calculated will be on the strength components of the tool. However, this result in lower scores being recorded hence the baseline status of the patients contribute to the initial PFIT-s scores recorded in patients. Although there was some improvement noted from the two measurements in our cohort, it showed participants in our group had a lower functional status level compared to the cohort from Australia, when comparing the PFIT-s measurements recorded for the two cohorts. The reason for this difference might be due to our inclusion criterion which was different. In our study we recruited our participants within 24 h of admission to the unit and intervention was started once physiological stability was achieved. In the study by Skinner et al. [34], participants were recruited in the study when they had stayed in the unit for more than five days. Also, in our study, the intervention was done only once per day for a period which ranged between 20 and 45 min, based on the functional capability of the patient until discharge from the unit whilst Skinner et al. [34] reported that intervention was given once while ventilated and increased to twice daily when participant was able to tolerate four consecutive hours free of the ventilator.

Higher PFIT-s scores at baseline and ICU discharge were found to be significantly associated with a reduced likelihood of discharge to a long-term acute care in the United States [43] and Denehy et al. [33] reported that a higher admission PFIT-s was predictive of a reduced acute care hospital length of stay. In these aforementioned studies, it showed that functional ability of the patients on admission is an important indicator to determine the long-term outcome and hence the need to improve the functional ability from the first day of admission into the unit.

### Limitations and recommendations

The study design used has some drawbacks which include, inability to make causal inferences and not able to control confounding variables. The sample size was quite small. It is recommended that this study should be replicated in a Randomised Controlled Trial with a larger sample size to detect significant differences in the results and be able to make a causal inference of the intervention.

The early mobility program intervention was only implemented at one centre, but future studies should use a number of hospitals over a longer period in order to ensure a larger sample size and be able to generalise the results. Patients should be followed up after discharge from the hospital for a long time to determine the change in outcomes over time.

Another limitation is not using severity of illness scores like APACHE score in ICU patients in Zimbabwe. The ICU clinicians should move towards using the severity of illness scores in order to monitor disease progression and also effect of each management.

Another limitation of the study was the retrospective registering of the trial on the *Pan African Clinical Trials Registry.*

## Conclusions

The results indicated that an early mobilisation program was feasible and safe in the management of critically ill in Zimbabwe. The implementation occurred with no adverse events recorded. The early mobilisation was done with the available resources and equipment in the unit without any additional equipment being purchased. The implementation of the intervention might be beneficial in improving long-term outcomes in the patients as the functional level of the patients had improved comparing the initial and final physical assessment measures using the tool. Specific inclusion and exclusion criteria can lead to a delayed early mobilisation activities in ICU patients.

## Abbreviations

HDU: High dependency unit
JREC: Joint research ethics committee
MRCZ: Medical Research Council of Zimbabwe
PFIT-s: Interval-scored physical function ICU test
